# MicroRNA Expression Profiling—Potential Molecular Discrimination of Papillary Thyroid Carcinoma Subtypes

**DOI:** 10.3390/biomedicines12010136

**Published:** 2024-01-09

**Authors:** Horatiu Silaghi, Laura Ancuța Pop, Carmen Emanuela Georgescu, Diana Muntean, Doinița Crișan, Patricia Silaghi, Ionela Lungu, Bogdana Adriana Nasui, Eva-H. Dulf, Cornelia Braicu, Ioana Berindan-Neagoe, Cristina Alina Silaghi

**Affiliations:** 1 Department of Surgery V, “Iuliu Hatieganu” University of Medicine and Pharmacy Cluj-Napoca, 8 Victor Babes Street, 400012 Cluj-Napoca, Romania; hsilaghi@yahoo.com; 2 Research Center for Functional Genomics, Biomedicine and Translational Medicine, “Iuliu Hatieganu” University of Medicine and Pharmacy Cluj-Napoca, 400337 Cluj-Napoca, Romania; braicucornelia@yahoo.com (C.B.); ioana.neagoe@umfcluj.ro (I.B.-N.); 3 Department of Endocrinology, “Iuliu Hatieganu” University of Medicine and Pharmacy Cluj-Napoca, 8 Victor Babes Street, 400012 Cluj-Napoca, Romania; c_e_georgescu@yahoo.com (C.E.G.); alinasilaghi@yahoo.com (C.A.S.); 4 Department of Pathology, Clinic Municipal Hospital Cluj-Napoca, Tăbăcarilor Street 11, 400139 Cluj-Napoca, Romania; drzamfirescu_diana@yahoo.com; 5 Department of Pathology, “Iuliu Hatieganu” University of Medicine and Pharmacy Cluj-Napoca, 6 Louis Pasteur Street, 400349 Cluj-Napoca, Romania; doinitacrisan@gmail.com; 6 Faculty of Medicine, “Iuliu Hatieganu” University of Medicine and Pharmacy Cluj-Napoca, 6 Louis Pasteur Street, 400349 Cluj-Napoca, Romania; patriciasilaghi01@yahoo.com; 7 Cardiomed Medical Center, 17 Republicii Street, 400015 Cluj-Napoca, Romania; ioni_2201@yahoo.com; 8 Department of Community Health, “Iuliu Hatieganu” University of Medicine and Pharmacy Cluj-Napoca, 6 Louis Pasteur Street, 400349 Cluj-Napoca, Romania; nasuibogdana@yahoo.ro; 9 Department of Automation, Faculty of Automation and Computer Science, Technical University of Cluj-Napoca, 28 Memorandumului Street, 400014 Cluj-Napoca, Romania; eva.dulf@aut.utcluj.ro

**Keywords:** biomarkers, diagnosis, miRNAs, thyroid cancer, papillary thyroid cancer, follicular variant of papillary thyroid cancer, tumourigenesis

## Abstract

Recent research has revealed the importance of miRNAs in the diagnosis and clinical evolution of papillary thyroid cancer (PTC). We aim to identify a specific miRNA profile that could differentiate between specific subtypes of PTC. Methods: In this cross-sectional study, total RNA was extracted from paraffin-embedded tissues of 43 patients, 17 with an infiltrative follicular variant of PTC (iFVPTC) and 26 with a conventional variant of PTC (cPTC). Nine miRNAs were evaluated using qRT-PCR technology and specific miRNA assays. Results: We found specific patterns for cPTC and iFVPTC, such as miRNA altered in both types of tumours (miR-146b-5p, miR-181a-5p, miR-221-3p, miR-21-5p and miR-222-3p) and two miRNAs significantly expressed only in cPTC (miR-20b-5p, miR-21-5p). The iFVPTC group presented strong and moderate correlations between miRNA expression and clinical data. miR-221-3p, miR-195-5p, miR-181-5p, miR-146b-5p and miR-222 were correlated with age, tumour size (TS) or lymph node metastases (N), while only miR-20b-5p, miR-195-5p and miR-181-5p were correlated with TS, N and age in the cPTC group. Conclusions: The present study allowed the identification of a signature of two miRNAs to confirm miRNA differences between the two histological subtypes of TC. Our results provide advances in the molecular diagnosis of TC and could help to improve the diagnostic performance of already existing molecular classifiers.

## 1. Introduction

Thyroid cancer (TC) represents presently the most prevalent endocrine malignancy, with an incidence that has increased rapidly in recent decades [1]. Follicular cell-derived tumours constitute the majority of thyroid neoplasms, and the most frequent type, papillary thyroid cancer (PTC), represents 85–90% of all TCs [2].

The fourth edition of the WHO Classification of Tumors of Endocrine Organs, published in 2017, linked histopathological classification and clinically appropriate risk stratification of thyroid tumours. Classical/conventional PTC (cPTC) has been shown to be the most common subtype of PTC (34%). Its molecular pathogenesis harbours several known mutational events, including point mutations in *BRAF* (50–77% of cPTC), especially *BRAF V600E*, and chromosomal rearrangements of the *RET* gene called *RET-PTC* rearrangement (8%), leading to the activation of the MAPK signalling pathway. The follicular variant of papillary thyroid carcinoma (FVPTC) has been described as the second most common histological subtype of PTC, constituting between 9% and 22.5% of all PTC cases [3,4] or accounting for up to 41% of PTC according to other authors [5]. FVPTC includes two subtypes: invasive encapsulated FVPTC (IEFV-PTC) and infiltrative FVPTC (iFVPTC). The non-invasive encapsulated follicular variant PTC has been reclassified since 2016 into the borderline tumour (non-malignant) category termed non-invasive follicular thyroid neoplasm with papillary-like nuclear features (NIFTP) and is considered to have a very low risk of adverse outcomes (negligible risk of recurrence or metastasis) [6].

FVPTC is generally associated with mutually exclusive mutations of *RAS or PAX8-PPARG* fusion alteration [7]. A recent review showed that all FVPTC accounted for 27% of PTC, of which 6% are the iFVPTC subtype, 4% are IEFV-PTC, and 17% are NIFTP [8]. Further, a retrospective review over 15 years showed different percentages of FVPTC of all PTC: 13.6% for NIFTP, 7.1% for IEFV-PTC subtype and 1.7% for iFVPTC [9]. A large multicentre study demonstrated that PTC and FVPTC have differentiated clinical implications and prognostic risks, which should lead to an individualised variant-specific-based treatment strategy for PTC. Recurrence and mortality of FVPTC were 9.1% and 0.6%, lower than for cPTC, where the values were 16.1% and 2.5%, respectively [10]. The overall prognosis of FVPTC was excellent and depended on the size of the tumour, the age of the patient, and the degree of angio-invasiveness, which predicts the risk of distant metastases. Other studies have shown conflicting results suggesting the opposite, more aggressive nature of FVPTC compared to cPTC, documenting a greater tendency for bone and lung metastases in FVPTC [11,12]. The rates of persistent and progressive disease were reported as 15% and 8% in FVPTC, respectively, suggesting a poorer prognosis than patients with cPTC and follicular TC (FCT) [13].

Recently, the 5th edition of the WHO Classification of Endocrine and Neuroendocrine Tumors has better integrated the pathological and molecular characteristics and the biological behaviour of thyroid tumours and divided them into several new categories [14]. IEFV-PTC is now considered a separate entity and no longer a subtype of PTC, having a *RAS-like* mutational and transcriptomic profile similar to that of follicular adenoma (FA) and FTC. Classic PTC and iFVPTC have *BRAFV600E-like* molecular profiles and are still considered subtypes of PTC [15]. Also, iFVPTC is associated with infiltrative growth and has a higher risk of lymph node metastasis, which is found in 65% of patients versus 7% for IEFV-PTC with capsular or vascular invasion [16].

MicroRNAs (miRNAs) are small, non-coding RNA molecules crucial in regulating gene expression. These molecules, typically composed of about 22 nucleotides, exert their influence by binding to the messenger RNA (mRNA) molecules, thereby modulating the translation of these mRNAs into proteins. The dysregulation of miRNAs can affect the expression of genes involved in key cellular processes, influencing the behaviour of TC cells. Identifying deregulated miRNAs promises to improve diagnostic accuracy or indicate TC progression, paving the way for developing novel and targeted therapeutic interventions. This exploration is also becoming of high interest for TC. Also, miRNAs have been implicated in various aspects of the TC, from its initiation to progression. Studying the altered miRNA profile in TC aims to identify and understand the specific deregulated miRNAs. This investigation is crucial for unravelling the intricacies of TC, potentially leading to the development of novel diagnostic tools and targeted therapeutic approaches. Studies carried out in recent years have highlighted the aberrant expression of miRNAs from the tissues of TC or peripheral blood of patients with TC and their influence on the activity of thyroid cancer-related regulation signalling pathways such as MAPK, PI3K, AKT, GSK-3β/β-catenin, Wnt, mTOR and NF-κB [17]. miRNAs are perceived as new, non-invasive and cost-effective diagnostic tests in PTC that would also be necessary in distinguishing between benign and malignant nodules, subsequently improving the selection of cases sent for surgery [17]. It is estimated that up to 50% of PTC surgeries are unnecessary, which can lead to potential treatment-related complications and an unjustified additional financial burden for the healthcare system. Several miRNAs have already been proposed in molecular panel tests to increase the sensitivity and specificity of fine needle aspiration biopsy (FNA) and to improve the preoperative diagnosis of malignancy in TC, especially in the case of indeterminate FNA (up to 30%). In addition to these benefits, miRNAs would contribute to the discrimination of different subtypes of TC, bringing new clarity to its clinical course [17,18].

To our knowledge, there are very few studies on specific miRNA expressions in different subtypes of TC. Therefore, this manuscript aims to identify a signature of miRNAs in iFVPTC versus cPTC and to detect possible differences in miRNA expression between the two histological types, which may bring additional evidence to refine the early diagnosis and the understanding of the distinct clinical course of cPTC and iFVPTC.

## 2. Materials and Methods

### 2.1. Altered miRNA Pattern in Thyroid Cancer

We used expression data from The Cancer Omics Atlas (TCOA) database, a comprehensive repository database that houses cancer omics data, including miRNA expression data [19]. TCOA offers the capability to access gene expression, somatic mutations, miRNA expression, and protein expression data for a specific molecule or cancer. Within the “Cancer” section of the platform, users have the option to choose a particular cancer type. All these results are connected to the chosen pathology and are contrasted with data from normal controls; data were accessed on 28 August 2023 (https://tcoa.cpu.edu.cn/).

#### Case Selection and Tumour Specimen Collection

This experiment has been conducted on retrospectively selected formalin-fixed, paraffin-embedded (FFPE) tissue specimens. This cross-sectional study included 43 patients with TC selected according to the following criteria: (1) thyroid surgery (total thyroidectomy) in a single tertiary hospital—Clinic Municipal Hospital Cluj-Napoca—in a 3-year period, performed by the same surgeon; (2) the tumour was cPTC or iFVPTC, stage I–III, with or without extrathyroidal extension/lymph node metastases; and (3) presence of both tumour and normal thyroid tissue to be analysed as a pair, in each patient. Pathological diagnoses were independently reconfirmed by two senior pathologists and reclassified following the 5th edition of World Health Organization Classification [15]. The tumour stage was assessed in line with the 8th TNM System of the American Joint Committee on Cancer (AJCC) classification. This retrospective study is carried out according to the Strengthening the reporting of observational studies in epidemiology (STROBE) guideline and represents a comparative observational study that meets the criteria of the checklist specified by the STROBE Statement as presented in Table S1 [20].

### 2.2. Macrodissection and RNA Extraction

FFPE tissue blocks were sectioned to produce 10 μm thick tissue sections. A 10 μm thick slide-mounted tissue section was stained with haematoxylin and eosin (H&E) as a template for macrodissection. Subsequently, it was reviewed by two certified pathologists to identify and confirm the histological diagnosis according to the current guidelines. Tumour region margins were marked with thin-tip sharpie on ten unstained 10 μm thick serial sections from the same tissue block, guided with the H&E-stained demarcated slide. Only the areas containing tumour cells inside the line were manually macrodissected from unstained sections and transferred to 1.5 mL Eppendorf tubes. In addition, non-tumour (normal) tissue was also collected from ten unstained slides for each patient. The genomic RNA was isolated from the FFPE tissue section using the RNeasy FFPE Kit (Qiagen, Germantown, MD, USA) following the manufacturer’s instructions. After the extraction, the RNA samples were quantified using a Nanodrop spectrophotometer (ThermoFisher, Waltham, MA, USA), and we obtained concentrations between 15 and 2000 ng/µL.

### 2.3. qRT-PCR Experiment

The RNA samples were used for cDNA synthesis using the TaqMan microRNA Reverse Transcription kit (Applied Biosystems, Waltham, MA, USA) starting from 50 ng of RNA/reaction and following the manufacturer’s instructions. We evaluated nine miRNAs (miR-20b-5p, miR-21-5p, miR-146b-5p, miR-181a-5p, miR-193-3p, miR-195-5p, miR-221-3p, miR-222-3p and miR-551b-3p) and miR-16-5p as the reference gene. The obtained cDNA was diluted six times with nuclease-free water and used in the qRT-PCR reaction. We prepared a mixture of 5.03 µL of ready-to-use TaqMan Fast Advance Master Mix (Applied Biosystems) and 0.47 µL of TaqMan microRNA primer, and we added 5.2 µL of cDNA for each of the selected miRNAs. From this mixture, we added 5 µL to two wells of the PCR plate. The PCR program used in the Viia 7 instrument was as follows: 1 cycle—2 min at 50 °C; 1 cycle—20 s at 95 °C; and 40 cycles at 95 °C for 1 s and 60 °C for 20 s in the FastMode. The obtained CT values were analysed using the ΔΔCT method, and the obtained results were imported into GraphPad Prism software v.10 for further analysis. For the analysis of the effect of multiple miRNAs, we used the Combiroc software (http://combiroc.eu) [21].

### 2.4. Functional Analysis

DIANA miRPath is a specialised tool designed to facilitate the identification of miRNA-targeted pathways through a friendly web interface (accessible at http://www.microrna.gr/miRPathv2, as of 24 August 2023) [22]. This tool serves as a valuable resource to analyse the potential interactions between miRNAs and pathways, aiding in the exploration of complex regulatory networks involving miRNAs and their associated biological functions. An additional miRNA–mRNA network interaction was generated using miRNET [23], shedding light on the regulatory dynamics within biological systems. Such networks can offer insights into how miRNAs modulate gene expression and potentially contribute to various biological processes and disease conditions.

## 3. Results

### 3.1. Altered miRNA Pattern in Thyroid Cancer—TCGA Data

**Figure 1:**
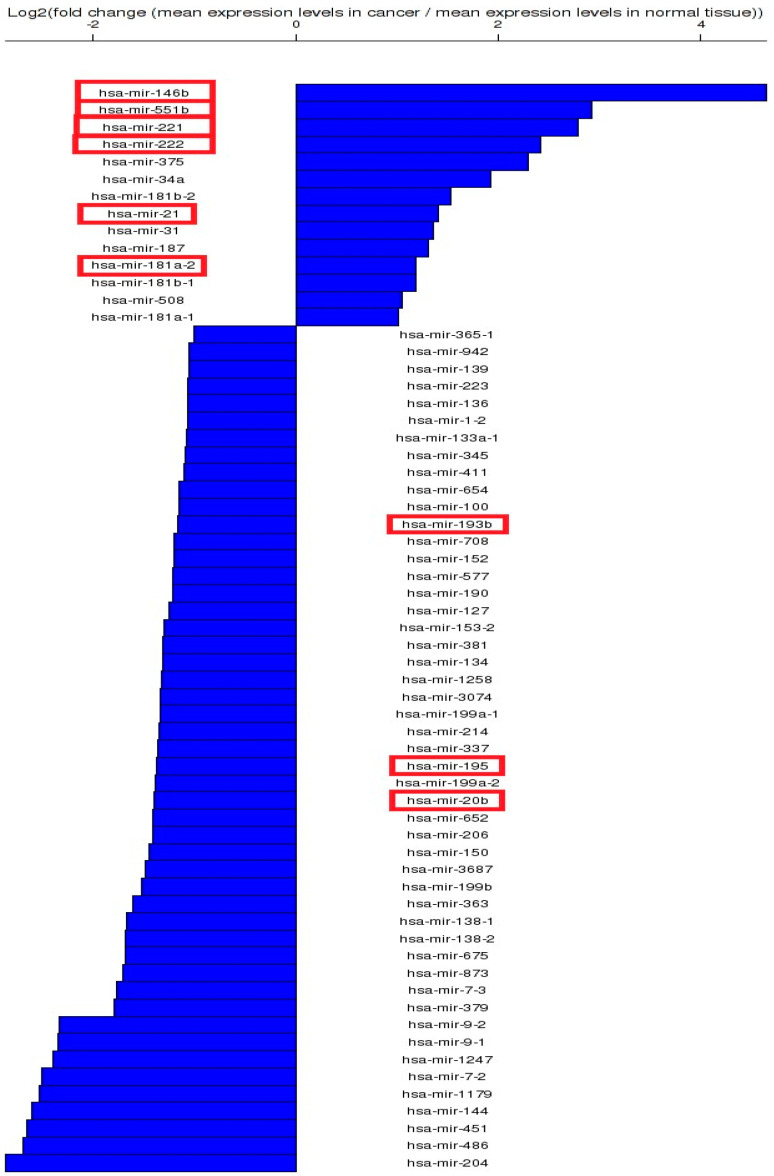
Graphical representation of top altered miRNA transcripts in TC, generated using the online portal TCOA (the red box presents the miRNAs selected for analysis).

MiRNA expression analysis was carried out using the TCOA public database, revealing 140 transcripts with an altered expression level, selecting a cut-off value |Fold change| > 1.5 and FDR (false discovery rate) *p*-value ≤ 0.05. Based on the data presented in Table S2, we selected for the validation of nine miRNAs from the top altered miRNA transcripts in TC, six being from the top overexpressed transcripts (miR-21-5p, miR-146b-5p, miR-181a-5p, miR-221-3p, miR-222-3p and miR-551b-3p) and three downregulated transcripts (miR-195-5p, miR-193b-3p, miR-20b-5p) (Figure 1).

### 3.2. Altered miRNA Pattern in Thyroid Cancer—Validation Using qRT-PCR

Clinical data of the 43 patients (17 with iFVPTC and 26 with cPTC) included in this study are presented in Table 1.

**Figure 2:**
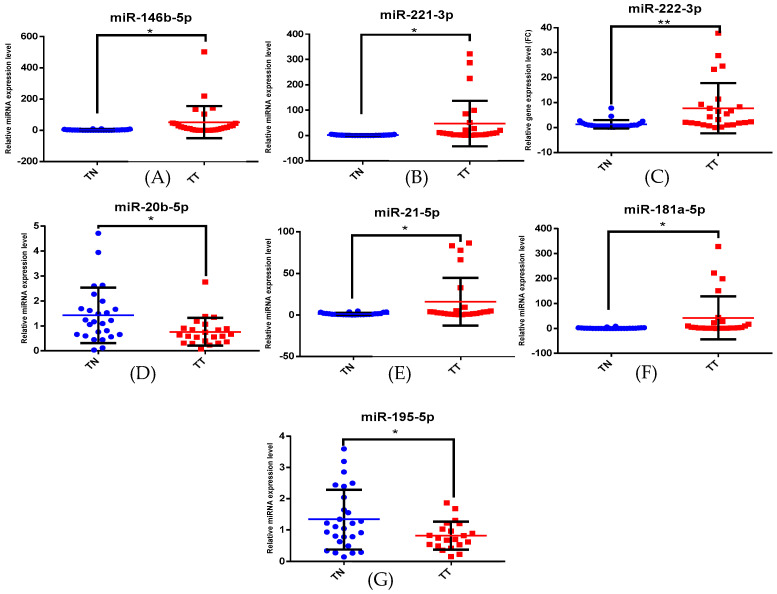
miRNA expression for (**A**) miR-146b-5p, (**B**) miR-221-3p, (**C**) miR-222-3p, (**D**) miR-20b-5p, (**E**) miR-21-5p, (**F**) miR-181a-5p and (**G**) miR-195-5p in cPTC patients. TT = tumour tissue; TN = normal tissue (* *p* < 0.05, ** *p* < 0.005).

**Figure 3:**
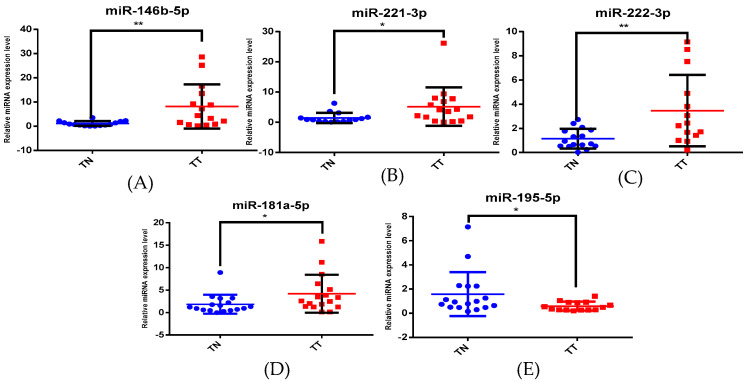
miRNA expression for (**A**) miR-146b-5p, (**B**) miR-221-3p, (**C**) miR-222-3p, (**D**) miR-181a-5p and (**E**) miR-195-5p in iFVPTC patients. TT = tumour tissue; TN = normal tissue (* *p* < 0.05, ** *p* < 0.005).

We obtained statistically significant levels for miR-146b-5p, miR-221-3p, miR-222-3p, miR-20b-5p, miR-21-5p, miR-181a-5p and miR-195-5p for the cPTC group (Figure 2) and for miR-146b-5p, miR-181a-5p, miR-195-5p, miR-221-3p and miR-222-3p for the iFVPTC group (Figure 3). We analysed the post hoc statistical power from the ClinCalc.com database [24] and found a power of >80% in our study.

**Figure 4:**
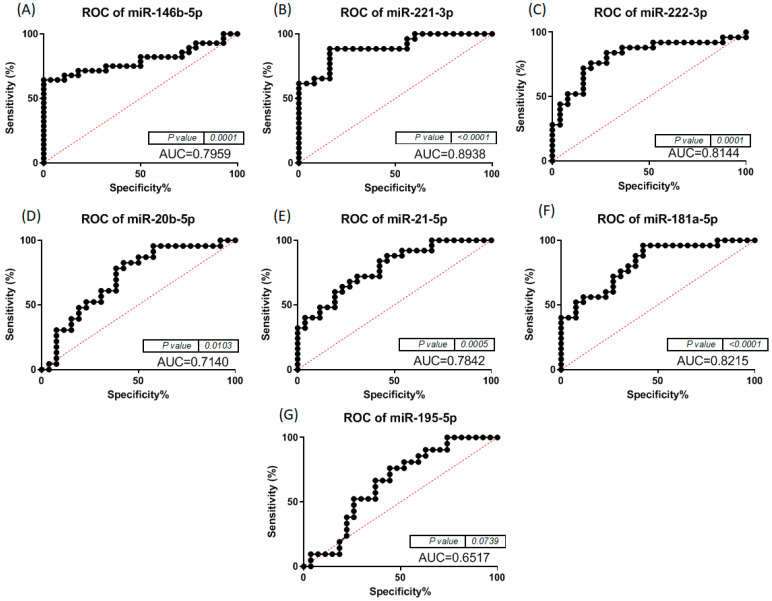
ROC curves for (**A**) miR-146b-5p, (**B**) miR-221-3p, (**C**) miR-222-3p, (**D**) miR-20b-5p, (**E**) miR-21-5p, (**F**) miR-181a-5p and (**G**) miR-195-5p in cPTC group.

**Figure 5:**
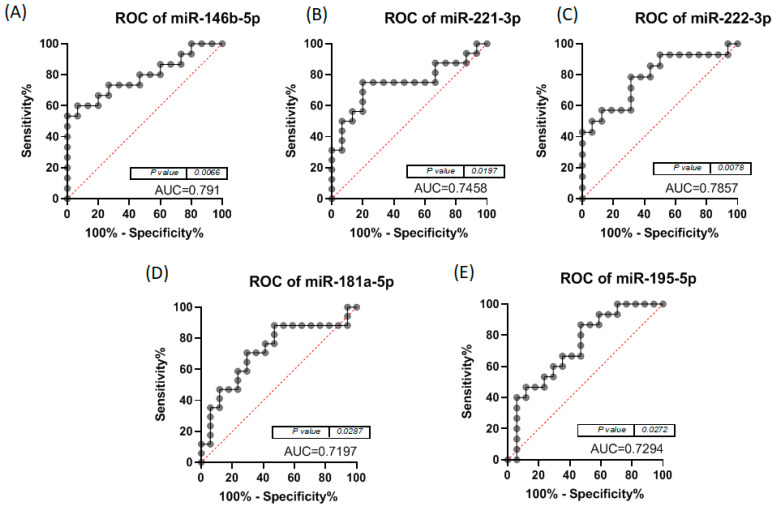
ROC curves for (**A**) miR-146b-5p, (**B**) miR-221-3p, (**C**) miR-222-3p, (**D**) miR-181a-5p and (**E**) miR-195-5p in iFVPTC group.

**Figure 6:**
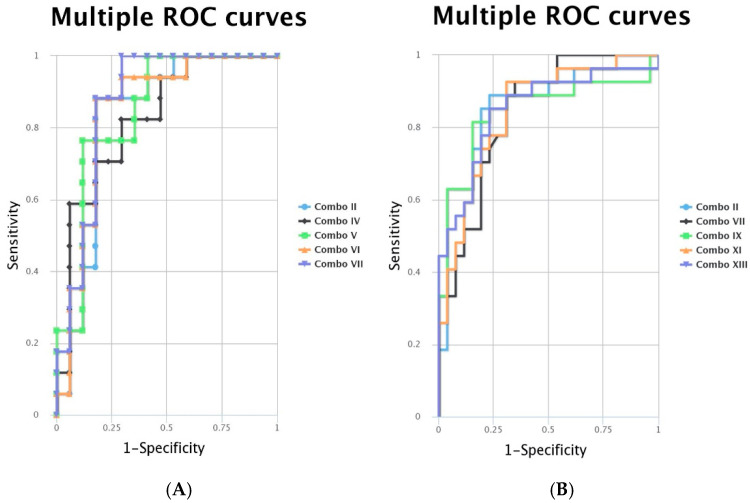
ROC curves for combinations of the tested miRNAs in (**A**) iFVPTC patients and (**B**) cPTC patients. Combinations of the selected miRNAs (presented in colours) are detailed in Table 2.

When analysing the ROC curves for the miRNAs with statistically significant expression alterations, we noted that for the comparison of normal to cPTC tumour samples, miR-221-3p, miR-181a-5p and miR-222-3p had the highest AUC, as can be seen in Figure 4. In the case of normal versus iFVTPC tumour samples, miR-146b-5p, miR-221-3p and miR-222-3p had the highest AUC, as seen in Figure 5. The *p*-value is indicated in Figure 4 and Figure 5 and shows significant values for most miRNAs. Due to the fact that the AUC in the single marker analysis was low, we also analysed the ROC curves for combinations of the tested markers. For iFVPTC, we observed better AUC values for several combinations of the tested markers, increasing up to 0.872, while for cPTC, the AUC was better than most of the single markers except for miR-221-3p. The exact combinations and their AUC are presented in Table 2, and Figure 6 shows the ROC curves for the specific combinations for the two groups of patients.

We also analysed the correlations between the tested miRNAs and observed strong and moderate direct correlations between miRNA expressions of most of the tested miRNAs in iFVPTC. Regarding the correlation between miRNA expression and clinical data, we observed a strong direct correlation between the presence or absence of lymph node metastases with miR-221-3p and a strong negative correlation between lymph node metastasis and miR-195-5p and miR-181-5p. A moderate positive correlation was observed between miR-146b-5p, miR-221-3p and miR-222-3p with age, T and tumour size. A moderate negative correlation was observed between miR-146b-5p with the presence or absence of lymph node metastases and miR-181-5p with age. All the correlations obtained are presented in Table 3.

In the case of cPTC patients, there were fewer correlations than in iFVPTC patients, and there were primarily strong direct correlations between the miRNA expressions of the tested miRNAs. In the case of the correlation between clinical data and miRNA expressions, there were strong direct correlations between miR-20b-5p and tumour size and between miR-195-5p and the presence of metastases in lymph nodes. miR-181-5p had a moderate positive correlation with lymphatic invasion and moderate negative correlation with the presence of metastases in the lymph node; mir-195-5p had a negative moderate correlation with age. The rest of the correlations were weak, as presented in Table 4.

Also, we investigated if there were significant modifications of the tested miRNAs between the two groups of patients, but no statistically significant differences were observed. We could only observe that miR-146b-5p, miR-181a-5p, miR-193-3p, miR-195-5p, miR-221-3p and miR-21-5p were downregulated in iFVPTC then in cPTC patients. We also found that miR-20b-5p, miR-222-3p and miR-551-5p were upregulated in iFVPTC then in cPTC patients.

### 3.3. Functional Analysis and Target Genes Identification for the Selected miRNAs

**Figure 7:**
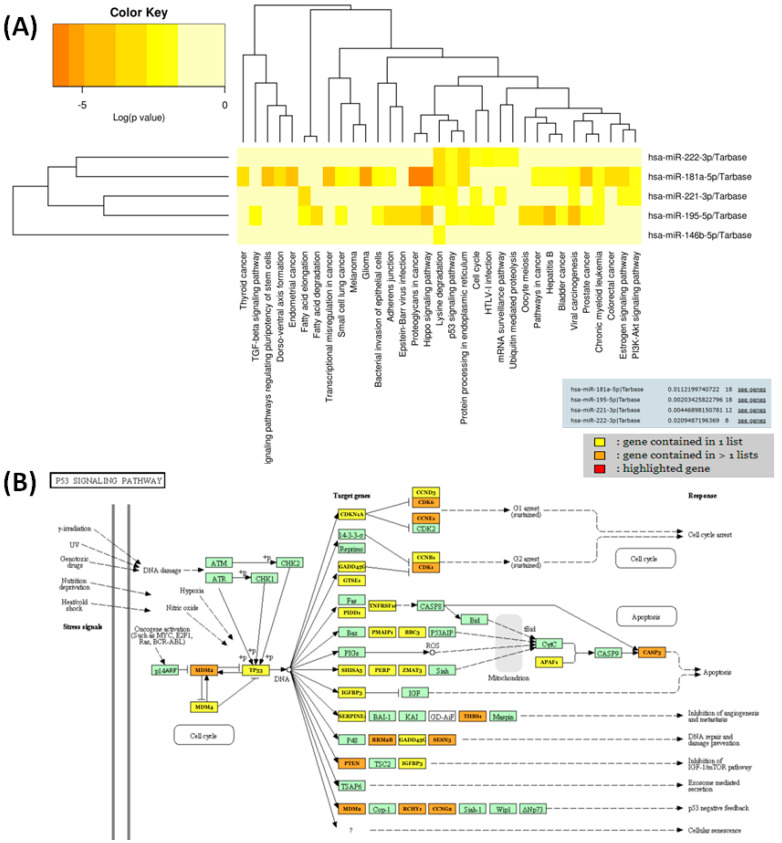
(**A**) Heatmaps were generated to visualise the notable pathways identified as significant by DIANA-miRPath (v.3.0) for the shared to common miRNA signature for the two subtypes of PTC. These heatmaps utilised clustering to arrange the pathways based on their significantly levels, with paths displayed along the x-axis and miRNAs along the y-axis. A colour code was applied to indicate the logarithmic (log) values of *p*-values. The most relevant interactions between predicted miRNAs and pathways were highlighted in red, (**B**) a graphical representation of the main target related to TP53 signalling for the selected miRNA panel.

Unravelling the functional roles and pinpointing the target genes of selected miRNAs hold significant implications. This knowledge not only contributes to our understanding of the molecular intricacies associated with these miRNAs but also opens up ways for potential therapeutic targets, diagnostic markers and the development of targeted interventions in various biological contexts. As was expected, TP53 was a key altered pathway involved in many cancers, often implicated in regulating cellular processes and stress responses, and also targeted by the analysed transcripts (Figure 7).

**Figure 8:**
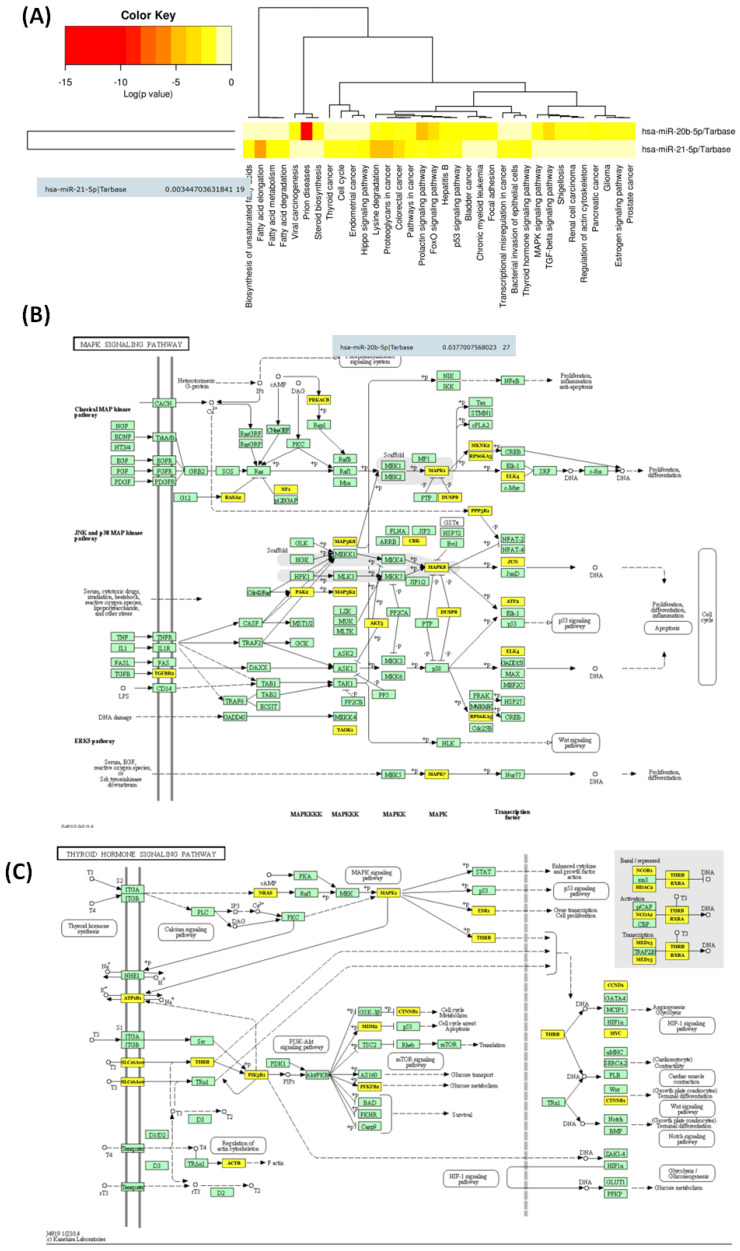
(**A**) Heatmaps were generated to visualise the notable pathways identified as significant by DIANA-miRPath (v.3.0) for the specific miRNA signature for cPTC. These heatmaps utilised clustering to arrange the pathways based on their significance levels, with paths displayed along the x-axis and miRNAs along the y-axis. A colour code was applied to indicate the logarithmic (log) values of *p*-values. The most relevant interactions between predicted miRNAs and pathways were highlighted in red, (**B**) a graphical representation of the main target related to MAPK signalling and (**C**) a Thyroid hormone signalling pathway for the selected miRNA panel.

This was the case for miR-20b, which was proven to be involved in the regulation of 27 genes related to MAPK signalling; meanwhile, miR-21-5p targets 19 genes related to the thyroid hormone signalling pathway (Figure 8).

## 4. Discussion

The present study identifies differences in miRNA expression between cPTC and iFVPTC versus adjacent normal tissue and between the two tissue subtypes. Specifically, analysis of miRNA expression levels in cPTC to adjacent normal tissue pointed out five significant upregulated miRNAs (miR-146b-5p, miR-181a-5p, miR-221-3p, miR-21-5p and miR-222-3p) and two significant downregulated miRNAs (miR-20b-5p, miR-195-5p) of all nine miRNAs analysed in this study. Of the seven miRNAs significantly expressed in cPTC, five are also significantly expressed in iFVPTC and vary in the same direction as in cPTC. Interestingly, we found two miRNAs significantly expressed only in cPTC and not in iFVPTC, namely miR-20b-5p downregulated and miR-21-5p upregulated versus normal tissue, which would represent a molecular mark that could differentiate between the two histological subtypes of PTC. The miRNA expression variation presented in this study is similar to that found in previous studies on TC, as well as presented in TCOA (Figure 1). We also observed that the main target for the selected five common miRNA panel is related to TP53 signalling (Figure 6B). Indeed, miRNAs can regulate key genes involved in the TP53 signalling pathway, influencing the intricate molecular mechanisms associated with cancer progression [25]. Understanding the intricate interplay between miRNAs and TP53 signalling in TC is essential for unravelling the molecular landscape of the disease. Dysregulation of these miRNAs contributes to altered TP53 signalling, impacting cell-cycle control, apoptosis and overall TC progression. Further research is needed to elucidate the specific roles of these miRNAs and their potential as therapeutic targets in TC treatment. Studies suggest that miR-20b dysregulation may play a role in TC progression by influencing key signalling pathways [26,27], including MAPK, as shown by the Diana miRpath analysis of this study, revealing 27 genes targeted by this transcript. miR-20b involvement in PTC is due to its regulatory impact on the MAPK signalling pathway [28] underscores its significance in the molecular landscape of PTC and its importance in differential diagnosis of TC subtypes. Previous studies showed that miR-20b has a tumour suppressor function in cancers and regulates essential biological processes such as cell proliferation, apoptosis, autophagy and migration, exerting its effects through diverse signalling pathways, including PI3K/AKT/mTOR, STAT, TGF-beta and ERK. Supporting this, Shubin Hong et al. suggest that downregulation of miR-20b displays tumour-suppressor functions in PTC, influencing tumour proliferation by targeting SOS1 and ERK2, inhibiting MAPK/ERK signalling pathway activity [28]. Also, the downregulation of miR 20b-5p in PTC could directly target DUXAP8 as non-coding RNA, influencing cell apoptosis. miRNA-20b could represent an essential factor in the initiation, progression and metastasis of PTC and may provide a potential therapeutic target for PTC [28,29,30].

Additionally, we found that miR-21-5p specifically targets 19 genes related to the thyroid hormone signalling pathway and unveils a noteworthy association with thyroid function and regulation. This finding suggests that miR-21-5p may play a role in modulating genes or components within the thyroid hormone signalling pathway, influencing the intricate regulatory mechanisms that govern thyroid function, especially the regulation of metabolism, growth and development. Understanding the specific target and its role in thyroid hormone signalling could provide insights into the broader implications of miR-21-5p in thyroid function and related disorders. Further exploration and validation of this interaction, both experimentally and through additional bioinformatics analyses, would contribute to a more comprehensive understanding of the regulatory networks involving miR-21-5p in the context of thyroid hormone signalling [27].

Equally, miR-21-5p, miR-146b-5p and miR-205-5p were the miRNAs most significantly overexpressed in PTC and seem to play a role in the carcinogenesis of PTC and, particularly, seem to correlate with its different biological behaviour [31]. miR-21-5p was significantly increased in TC cell lines and was negatively correlated with its target gene recombinant sclerostin domain-containing protein 1 SOSTDC1 (r = −0.24, *p* < 0.01), affecting the activities of PI3K/Akt and MAPK/ERK, thereby inhibiting the apoptosis of TC cells and promoting cell proliferation and migration [32]. Also, plasma exosomal miR-21 and miR-181a differentiate FTC from PTC with 100% sensitivity and 77% specificity [33].

Thus, the results of our study relating to the significant variation in miR-21-5p and miR-20b-5p might add some additional data to the candidate miRNA biomarkers in the distinction of cPTC versus iFVPTC.

The ROC curves visible in Fig 3 show an AUC over 0.8 for miR-181a-5p, miR-221-3p and miR-222-3p, indicating a strong association between cPTC and the three miRNAs. An AUC between 0.6 and 0.8 were found for miRNAs significantly expressed in iFVPTC, shown in Figure 5. Additionally, we also found that the simultaneous presence of miRNAs, such as, for example, the selected combination of miR-222-3p + miR-195-5p, significantly increased the AUC in iFVPTC (Table 2), while in cPTC, the selected miRNA combinations slightly increased the AUC. These results could support the hypothesis that certain combinations of the miRNAs reported in this study could enhance the sensitivity and specificity of cPTC and iFVPTC diagnosis. Still, the ROC curves for combinations of miRNAs tested in patients with iFVPTC and in patients with cPTC are shown in Figure 6 and allow a clearer visualisation of the benefit of the combinations of miRNAs for the accuracy of diagnosis of cPTC and iFVPTC, respectively.

Generally, miRNAs are emerging as a promising tool in the discovery of biomarkers for screening/early diagnosis, patient stratification and prognostic indicators, and therapeutic target purposes [34]. The identified microRNAs, which are most consistently reported in this study, may be potential diagnostic/prognostic biomarkers and therapeutic targets. Our results align with previous reports, as the significantly expressed miRNAs presented in this study, are confirmed in TC in other articles, including both circulating miRNAs and miRNAs from TC tissue. So, a study on 19 microRNA expression profiling comparing miRNA expression data of TC and associated normal tissues observed 138 aberrantly expressed miRNAs. The identified miRNAs, which are most consistently reported in PTC (upregulated miR-221-5p, miR-222-5p, miR-34a-5p, miR-146b-5p, miR-21-5p) may be potential diagnostic/prognostic biomarkers and therapeutic targets [34]. Other studies showed that miR-221 and miR-222 are upregulated in PTC compared with normal thyroid tissue or follicular adenoma and hyperplastic nodules [35,36], and both are associated with an increased risk of recurrence of PTC through the presence of capsule invasion, vascular invasion or lymph node metastasis [37,38]. We previously underlined the beneficial prognostic role of miRNAs in PTC. In a recent meta-analysis of 18 studies, we showed the potential role as screening/prognostic biomarkers of miR-146b, miR-221 and miR-222 for recurrent PTC [39]. If we were to analyse circulating miRNA, miR-146, miR-221 and miR-222 would be frequently encountered in the literature and present the strongest association with PTC. It was observed that in some studies, miR-221-3p showed 100% specificity and miR-146b showed 94.3% sensitivity, while the combination of miR-146b, miR-187, miR-138, miR- 375, miR-222-3p, miR-151a-3p and thyroid ultrasound had the highest AUC [40,41].

The prevalence of high-risk parameters, including extrathyroidal invasion, lymph node metastasis, stages III/IV, disease recurrence, mortality and the use (need) of radioiodine treatment is lower in iFVPTC compared with cPTC. We analysed several clinical parameters, and we found, for iFVPTC, a direct strong correlation between the presence or absence of lymph node metastases (N) with miR-221-3p and a negative strong correlation between N with miR-195-5p and miR-181-5p. A moderate positive correlation was observed between miR-146b-5p, miR-221-3p and miR-222-3p with age, T and tumour size. A moderate negative correlation was observed between miR-146b-5p with the presence or absence of lymph node metastases. In cPTC, tumour dimensions are strongly directly correlated with miR-20b-5p. We found strong direct correlations between N and miR-195-5p and moderate indirect correlation between miR-195-5p with age. In the present study, the numerous correlations between miRNAs and between miRNAs and clinical parameters show the undoubted involvement of miRNAs in the clinical evolution of PTC, more precisely, a different and specific involvement of miRNAs in the evolution of certain clinical parameters in cPTC versus iFVPTC. Thus, miRNAs along with other molecules such as identified differentially expressed proteins could have a role as potential tissue biomarkers for PTC metastases [42].

One of the limitations of this study is the small number of samples analysed. Further studies are needed to validate these results in a larger population-based cohort and to better understand the involvement of these miRNAs in the clinical behaviour of the two histological subtypes.

## 5. Conclusions

Our data revealed the importance of studying miRNAs as we were able to identify a common miRNA signature (miR-146b-5p, miR-181a-5p, miR-221-3p, miR-222-3p and miR-195-5p) and two transcripts (miR-20b-5p downregulated, miR-21-5p upregulated) specific to cPTC. This confirmation of miRNA differences carries potential pathophysiological implications, offering a deeper understanding of the intricate molecular mechanisms at play. The present study confirms miRNA differences in the two histological subtypes of PTC. It suggests that identifying the miRNA expression profile could shed light on the molecular mechanism and bring more clarity to understanding the different clinical evolutions of cPTC and iFVPTC. Moreover, we highlighted that some miRNAs, whose utility in TC has been poorly investigated until now, could serve as histotype-specific miRNAs. Our results provide advances in the molecular diagnosis of TC and could help to improve the diagnostic performance of already existing molecular classifiers and could support the selection of miRNA targets for setting up new non-invasive diagnostic panels to be used in precision medicine.

Further research is essential to confirm these results in larger patient samples, to validate and delineate the specific targets and mechanisms by which the studied miRNAs modulate key pathways in TC, paving the way for their applications in clinical practice and offering insights that could potentially be leveraged for therapeutic advancements in TC management.

## Figures and Tables

**Table 1:** Clinical data of the enrolled patients.

	cPTC	iFVPTC
Age mean (years)	57.69	52.05
Age range (years)	44–74	28–74
Females	14	14
Males	12	3
T1	14	9
T2	9	3
T3	3	5
N0	3	2
N1	6	2
Nx	17	13
Mx	26	17
Tumour size mean (mm)	19.42	22.58

cPTC = conventional variant PTC; iFVPTC = infiltrative follicular variant of PTC.

**Table 2:** AUC values for combinations of the selected miRNAs.

Combo iFVPTC	Markers	AUC	Combo cPTC	Markers	AUC
II	miR-146-5p + miR-20b-5p	0.83	II	miR-221-3p + miR-20b-5p	0.858
IV	miR-222-3p + miR-20b-5p	0.827	VII	miR-20b-5p + miR-181a-5p	0.843
V	miR-222-3p + miR-195-5p	0.848	IX	miR-221-3p + miR222-3p + miR-20b-5p	0.846
VI	miR-146-5p + miR-222-3p + miR-20b-5p	0.848	XI	miR-221-3p + miR-20b-5p + miR-195-5p	0.846
VII	miR-222-3p + miR-20b-5p + miR-195-5p	0.872	XIII	miR-221-3p + miR222-3p + miR-20b-5p + miR-195-5p	0.852

**Table 3:** Correlation between the expression levels of tested miRNAs in the iFVPTC group; values are for the correlation coefficient (red = strong correlation, blue = moderate correlation, green = weak correlation, positive value = direct correlation, negative value = inverse correlation). T = size of the TC and whether it has invaded nearby tissue, N = nearby (regional) metastatic lymph nodes.

iFVPTC	miR-146b-5p	miR-221-3p	miR-222-3p	miR-181-5p	miR-195-5p	Age	T	N	Tumour Size (mm)	Lymphatic Invasion	Multifocality
miR-146b-5p		0.999	0.998	−0.002	0.762	0.324	0.348	−0.383	0.357	−0.066	−0.163
miR-221-3p	0.999		0.997	0.012	0.761	0.320	0.355	0.685	0.358	−0.066	−0.169
miR-222-3p	0.998	0.997		−0.034	0.769	0.319	0.365	−0.125	0.383	−0.066	−0.184
miR-181-5p	−0.002	0.012	−0.034		−0.095	−0.421	0.066	−0.663	−0.002	−0.179	0.011
miR-195-5p	0.762	0.761	0.769	−0.095		0.226	0.164	−0.844	0.197	−0.166	−0.149
Age	0.324	0.320	0.319	−0.421	0.226		0.208	0.989	0.128	0.071	0.411
T	0.348	0.355	0.365	0.066	0.164	0.208		0.577	0.952	−0.218	−0.223
N	−0.383	0.685	−0.125	−0.663	−0.844	0.989	0.577		0.455	0.577	
Tumour size (mm)	0.357	0.358	0.383	−0.002	0.197	0.128	0.952	0.455		−0.193	−0.349
Lymphatic invasion	−0.066	−0.066	−0.066	−0.179	−0.166	0.071	−0.218	0.577	−0.193		−0.185
Multifocality	−0.163	−0.169	−0.184	0.011	−0.149	0.411	−0.223		−0.349	−0.185	
Radioiodine therapy	0.166	0.166	0.169	0.28	−0.115	−0.201	0.563		0.620	0.161	−0.604

**Table 4:** Correlation of the expression levels of tested miRNAs in the cPTC group; values are for the Pearson correlation coefficient (red = strong correlation, blue = moderate correlation, green = weak correlation, positive value = direct correlation, negative value = inverse correlation). T = size of the TC and whether it has invaded nearby tissue, N = nearby (regional) metastatic lymph nodes.

cPTC	miR-146b-5p	miR-221-3p	miR-222-3p	miR-20b-5p	miR-21-5p	miR-181-5p	miR-195-5p	Age	T	N	Tumour Size (mm)	Lymphatic Invasion	Multifocality
miR-146b-5p		0.65	1.00	−0.31	0.88	0.65	−0.21	0.11	0.12	0.00	0.22	−0.07	−0.11
miR-221-3p	0.65		0.68	−0.30	0.73	0.73	−0.18	−0.11	0.14	0.16	0.13	0.20	0.02
miR-222-3p	1.00	0.68		−0.24	0.65	0.57	−0.18	0.08	0.12	−0.07	0.22	−0.06	−0.12
miR-20b-5p	−0.31	−0.30	−0.24		−0.38	−0.02	−0.02	0.20	0.10	0.19	0.62	−0.09	−0.07
miR-21-5p	0.88	0.73	0.65	−0.38		0.55	−0.16	−0.09	0.02	−0.17	−0.09	0.15	0.17
miR-181-5p	0.65	0.73	0.57	−0.02	0.55		−0.30	−0.12	−0.08	−0.30	−0.01	0.43	−0.09
miR-195-5p	−0.21	−0.18	−0.18	−0.02	−0.16	−0.30		−0.44	−0.01	0.64	−0.04	−0.01	0.00
Age	0.11	−0.11	0.08	0.20	−0.09	−0.12	−0.44		−0.09	−0.58	−0.11	−0.11	0.20
T	0.12	0.14	0.12	0.10	0.02	−0.08	−0.01	−0.09		0.46	0.52	0.42	−0.13
N	0.00	0.16	−0.07	0.19	−0.17	−0.30	0.64	−0.58	0.46		0.44	0.38	0.16
Tumour size(mm)	0.22	0.13	0.22	0.62	−0.09	−0.01	−0.04	−0.11	0.52	0.44		−0.13	−0.30
Lymphatic invasion	−0.07	0.20	−0.06	−0.09	0.15	0.43	−0.01	−0.11	0.42	0.38	−0.13		0.22
Multifocality	−0.11	0.02	−0.12	−0.07	0.17	−0.09	0.00	0.20	−0.13	0.16	−0.30	0.22	

## Data Availability

Data are contained within the article.

## Supplementary Materials

The following supporting information can be downloaded at: https://www.mdpi.com/article/10.3390/biomedicines12010136/s1, Table S1: STROBE Statement—Checklist of items that should be included in reports of cross-sectional studies, Table S2: Altered miRNA panel in thyroid cancer.
