# Supplementary material for: MicroRNA Expression Profiling—Potential Molecular Discrimination of Papillary Thyroid Carcinoma Subtypes
Source: Biomedicines. 2024 Jan 9;12(1):136. doi: 10.3390/biomedicines12010136 (PMC10813560; doi:10.3390/biomedicines12010136)
Supplement: Supplementary file 1 [file biomedicines-12-00136-s001.zip › Supplimentary Table 2 H_Silaghi.pdf]

Table S2. Altered miRNA panel in thyroid cancer.

Gene	P-value	Log2(Fold change)	FDR(q-value)	Fold Change
hsa-mir-146b	1.08E-37	4.649519	1.41E-35	25.09832
hsa-mir-551b	2.54E-28	2.926408	1.21E-26	7.602153
hsa-mir-221	2.77E-39	2.783308	4.84E-37	6.884292
hsa-mir-222	1.12E-33	2.415093	7.82E-32	5.33354
hsa-mir-375	1.14E-12	2.289376	1.32E-11	4.888445
hsa-mir-34a	2.43E-53	1.919589	1.27E-50	3.783154
hsa-mir-181b-2	1.34E-34	1.532124	1.08E-32	2.892113
hsa-mir-21	6.87E-23	1.413058	1.94E-21	2.66301
hsa-mir-31	1.07E-06	1.355889	5.67E-06	2.559547
hsa-mir-187	1.38E-09	1.308978	1.11E-08	2.477659
hsa-mir-181a-2	6.24E-23	1.191311	1.81E-21	2.283602
hsa-mir-181b-1	7.56E-28	1.189786	3.44E-26	2.281188
hsa-mir-508	5.24E-09	1.057	3.83E-08	2.080601
hsa-mir-181a-1	1.69E-23	1.013686	5.21E-22	2.019064
hsa-mir-514-2	1.55E-09	0.953174	1.23E-08	1.936128
hsa-mir-934	1.15E-10	0.91847	1.06E-09	1.89011
hsa-mir-514-3	1.04E-08	0.887792	7.35E-08	1.850342
hsa-mir-514-1	9.79E-09	0.887466	6.96E-08	1.849924
hsa-mir-509-3	3.55E-09	0.868256	2.69E-08	1.825454
hsa-mir-509-1	6.60E-10	0.846276	5.52E-09	1.797854
hsa-mir-503	4.70E-06	0.82169	2.26E-05	1.767475
hsa-mir-96	3.85E-10	0.800295	3.27E-09	1.741458
hsa-mir-3065	1.80E-05	0.788426	8.11E-05	1.727188
hsa-mir-509-2	2.58E-08	0.771787	1.72E-07	1.707383
hsa-mir-183	3.74E-07	0.765225	2.08E-06	1.699635
hsa-mir-181d	1.41E-08	0.644836	9.76E-08	1.563561
hsa-mir-182	1.12E-05	0.617765	5.12E-05	1.534496
hsa-mir-1274b	0.001276	-0.58837	0.004305	0.665093
hsa-mir-218-1	1.83E-08	-0.59523	1.25E-07	0.661937
hsa-mir-624	1.18E-17	-0.5991	2.16E-16	0.660164
hsa-mir-376c	3.69E-07	-0.60372	2.06E-06	0.658053
hsa-mir-34c	2.91E-06	-0.60576	1.44E-05	0.657127
hsa-mir-101-2	6.28E-11	-0.60708	6.09E-10	0.656523
hsa-mir-574	2.46E-15	-0.62023	3.91E-14	0.650569
hsa-mir-1291	7.96E-19	-0.62085	1.60E-17	0.650288
hsa-mir-153-1	3.98E-20	-0.6323	8.86E-19	0.645147
hsa-mir-26b	1.42E-13	-0.63485	1.83E-12	0.644008
hsa-mir-30c-2	2.82E-10	-0.64142	2.45E-09	0.641081
hsa-mir-29c	1.02E-10	-0.651	9.61E-10	0.636839
hsa-mir-1270-2	2.01E-10	-0.65132	1.80E-09	0.636698

hsa-mir-10b	4.89E-07	-0.65644	2.68E-06	0.634443
hsa-mir-582	1.16E-09	-0.65652	9.46E-09	0.634407
hsa-mir-369	1.85E-06	-0.65846	9.51E-06	0.633552
hsa-mir-382	8.69E-05	-0.66448	0.000344	0.630915
hsa-mir-25	2.99E-26	-0.66734	1.20E-24	0.629667
hsa-mir-370	3.61E-07	-0.66845	2.03E-06	0.629183
hsa-mir-3653	4.44E-09	-0.67179	3.31E-08	0.627727
hsa-mir-342	2.87E-09	-0.67314	2.22E-08	0.627139
hsa-mir-484	1.61E-12	-0.67546	1.81E-11	0.626131
hsa-let-7c	2.74E-12	-0.69431	2.93E-11	0.618006
hsa-mir-3676	2.44E-05	-0.70332	0.000105	0.614155
hsa-mir-210	5.53E-05	-0.71029	0.000223	0.611199
hsa-mir-126	5.02E-09	-0.7106	3.69E-08	0.611067
hsa-mir-584	2.70E-12	-0.71138	2.91E-11	0.610737
hsa-mir-3607	2.57E-05	-0.71966	0.000109	0.607242
hsa-mir-143	3.77E-07	-0.72083	2.09E-06	0.606749
hsa-mir-874	2.17E-06	-0.72296	1.11E-05	0.605853
hsa-mir-3912	1.20E-18	-0.72383	2.37E-17	0.605488
hsa-mir-1270-1	2.52E-12	-0.72448	2.74E-11	0.605214
hsa-mir-130a	6.11E-11	-0.72555	5.97E-10	0.604767
hsa-mir-16-2	3.40E-18	-0.72925	6.47E-17	0.603217
hsa-mir-99a	5.11E-16	-0.73128	8.49E-15	0.602367
hsa-mir-154	2.59E-09	-0.73766	2.02E-08	0.599713
hsa-mir-374b	5.57E-27	-0.74707	2.43E-25	0.595814
hsa-mir-148a	2.88E-08	-0.74916	1.89E-07	0.594949
hsa-let-7b	1.11E-13	-0.75243	1.45E-12	0.593602
hsa-mir-30a	3.30E-15	-0.75907	5.15E-14	0.590875
hsa-mir-26a-2	8.27E-29	-0.7742	4.32E-27	0.584715
hsa-mir-1275	1.28E-12	-0.77468	1.47E-11	0.584516
hsa-mir-326	1.53E-09	-0.7781	1.22E-08	0.583134
hsa-mir-196b	3.80E-07	-0.78684	2.09E-06	0.579614
hsa-mir-758	2.47E-06	-0.80328	1.24E-05	0.573043
hsa-mir-145	2.67E-10	-0.8107	2.35E-09	0.570105
hsa-mir-876	2.90E-31	-0.82225	1.69E-29	0.565561
hsa-mir-3652	8.72E-18	-0.82572	1.63E-16	0.564199
hsa-mir-425	2.29E-19	-0.84561	4.90E-18	0.556476
hsa-mir-3130-1	8.34E-09	-0.84578	5.97E-08	0.556411
hsa-mir-142	2.34E-05	-0.86106	0.000102	0.550549
hsa-mir-193a	1.20E-14	-0.86779	1.74E-13	0.547987
hsa-mir-133b	3.17E-09	-0.87901	2.44E-08	0.54374
hsa-mir-130b	1.37E-10	-0.88062	1.25E-09	0.543134
hsa-mir-215	4.80E-21	-0.88629	1.12E-19	0.541005
hsa-mir-10a	9.58E-12	-0.8895	9.83E-11	0.539799
hsa-mir-106a	4.99E-24	-0.89796	1.74E-22	0.536647
hsa-mir-28	2.31E-33	-0.92193	1.42E-31	0.527803

hsa-mir-455	2.05E-12	-0.92567	2.28E-11	0.526436
hsa-mir-18a	1.36E-22	-0.96061	3.65E-21	0.513838
hsa-mir-218-2	2.12E-17	-0.97187	3.83E-16	0.509844
hsa-mir-497	3.69E-16	-0.9788	6.23E-15	0.507402
hsa-mir-365-2	8.85E-23	-0.99493	2.44E-21	0.501759
hsa-mir-365-1	2.59E-23	-1.0098	7.73E-22	0.496616
hsa-mir-942	3.94E-30	-1.0485	2.17E-28	0.483471
hsa-mir-139	2.87E-13	-1.05144	3.61E-12	0.482485
hsa-mir-223	3.81E-13	-1.06145	4.69E-12	0.47915
hsa-mir-136	2.73E-07	-1.06321	1.56E-06	0.478567
hsa-mir-1-2	9.80E-10	-1.0714	8.07E-09	0.475858
hsa-mir-133a-1	3.05E-10	-1.07755	2.64E-09	0.473834
hsa-mir-345	2.41E-10	-1.08837	2.13E-09	0.470293
hsa-mir-411	3.91E-15	-1.10702	6.02E-14	0.464253
hsa-mir-654	1.26E-09	-1.14701	1.02E-08	0.45156
hsa-mir-100	6.20E-21	-1.14875	1.41E-19	0.451016
hsa-mir-193b	1.47E-16	-1.16368	2.56E-15	0.446373
hsa-mir-708	4.23E-12	-1.19891	4.43E-11	0.435603
hsa-mir-152	1.10E-21	-1.19927	2.68E-20	0.435496
hsa-mir-577	1.11E-12	-1.20805	1.31E-11	0.432853
hsa-mir-190	3.03E-22	-1.21533	7.73E-21	0.430674
hsa-mir-127	7.22E-07	-1.2443	3.87E-06	0.422114
hsa-mir-153-2	9.41E-24	-1.30239	2.98E-22	0.405455
hsa-mir-381	2.29E-11	-1.30756	2.30E-10	0.404003
hsa-mir-134	1.97E-08	-1.31652	1.33E-07	0.401501
hsa-mir-1258	9.96E-55	-1.32707	1.04E-51	0.398577
hsa-mir-3074	1.19E-26	-1.3365	5.00E-25	0.395979
hsa-mir-199a-1	4.50E-13	-1.34076	5.47E-12	0.394814
hsa-mir-214	6.20E-16	-1.35304	1.01E-14	0.391467
hsa-mir-337	1.02E-13	-1.36156	1.35E-12	0.389162
hsa-mir-195	8.75E-29	-1.37401	4.36E-27	0.385817
hsa-mir-199a-2	5.60E-14	-1.39058	7.60E-13	0.381411
hsa-mir-20b	4.71E-19	-1.39309	9.66E-18	0.380749
hsa-mir-652	4.31E-37	-1.4049	5.01E-35	0.377645
hsa-mir-206	1.97E-14	-1.40871	2.78E-13	0.376647
hsa-mir-150	5.93E-11	-1.4427	5.85E-10	0.367879
hsa-mir-3687	1.12E-34	-1.48218	9.76E-33	0.357947
hsa-mir-199b	9.15E-16	-1.52493	1.47E-14	0.347497
hsa-mir-363	2.06E-34	-1.60649	1.54E-32	0.328396
hsa-mir-138-1	7.81E-24	-1.67435	2.64E-22	0.313307
hsa-mir-138-2	8.93E-25	-1.67623	3.34E-23	0.312898
hsa-mir-675	2.24E-18	-1.68093	4.35E-17	0.311881
hsa-mir-873	1.83E-38	-1.70656	2.74E-36	0.306389
hsa-mir-7-3	2.27E-21	-1.7616	5.39E-20	0.29492
hsa-mir-379	8.89E-13	-1.79098	1.06E-11	0.288976

hsa-mir-9-2	1.26E-35	-2.33389	1.20E-33	0.198348
hsa-mir-9-1	5.73E-36	-2.35075	5.99E-34	0.196044
hsa-mir-1247	1.50E-19	-2.39288	3.26E-18	0.190402
hsa-mir-7-2	8.58E-24	-2.50782	2.80E-22	0.17582
hsa-mir-1179	1.32E-33	-2.53595	8.62E-32	0.172426
hsa-mir-144	1.35E-42	-2.60976	4.70E-40	0.163826
hsa-mir-451	4.69E-40	-2.64883	9.81E-38	0.159449
hsa-mir-486	1.46E-41	-2.69182	3.83E-39	0.154769
hsa-mir-204	1.39E-22	-2.86871	3.63E-21	0.136909
